# Effects of rice straw fermented with spent *Pleurotus sajor-caju* mushroom substrates on milking performance in Alpine dairy goats

**DOI:** 10.5713/ab.21.0340

**Published:** 2022-01-05

**Authors:** Geng-Jen Fan, Mei-Hsing Chen, Churng-Faung Lee, Bi Yu, Tzu-Tai Lee

**Affiliations:** 1 Animal Industry Division, Livestock Research Institute (LRI), Council of Agriculture (COA), Hsinhua, Tainan 712009, Taiwan; 2 Department of Animal Science, National Chung Hsing University (NCHU), Taichung 402204, Taiwan; 3 Plant Pathology Division, Taiwan Agricultural Research Institute, Taichung City 413008, Taiwan; 4 Deputy director office, LRI, Hsinhua, Tainan 712009, Taiwan; 5 The iEGG and Animal Biotechnology Center, National Chung Hsing University, Taichung, 402, Taiwan; 6 Smart Sustainable New Agriculture Research Center (SMARTer), National Chung Hsing University, Taichung, 402, Taiwan

**Keywords:** Fermented Rice Straw, Lactating Dairy Goat, Rice Straw, Spent Mushroom Substrates

## Abstract

**Objective:**

To improve the feeding value of rice straw (RS), this study evaluated the potential of rice straw fermented with *Pleurotus sajor-caju* (FRS) as dairy goat feed.

**Methods:**

Spent *Pleurotus sajor-caju* mushroom substrate was used as fungi inoculum to break the lignocellulose linkage of rice straw, which was solid-fermented at 25°C to 30°C for 8 weeks. The ruminal degradation of pangolagrass hay (PG), FRS, and RS were measured *in situ* for 96 hours in three dry Holstein cows, respectively. Effect of fungi fermented RS on milking performance was studied in feeding trials. A total of 21 Alpine goats a trial were divided into 3 groups: a control group in which PG accounted for 15% of the diet dry matter, and FRS or RS was used to replace the PG in the control group. Goats were fed twice a day under two 28-day trial in individual pens. Meanwhile, a 3×3 Latin square trial (14 days/period) was conducted to study the rumen digestion of three diets by using three fistulated dry goats. Rumen contents were collected for metabolite analyses every one to three hours on the last two days.

**Results:**

*In situ* study showed that fermentation could elevate the rumen degradable fraction and effective degradability of RS (p<0.05). Effective degradability of FRS dry matter was significantly increased from 29.5% of RS to 41.7%. Lactating trial results showed that dry matter intake and milk yield in the PG group and FRS group were similar and higher than those in RS group (p<0.05). The concentration of propionic acid and total volatile fatty acid in the RS group tended to be lower than those in PG group (p<0.10). There were no differences in rumen pH value and ammonia nitrogen level among the groups tested.

**Conclusion:**

Fermentation of rice straw by spent *Pleurotus sajor-caju* mushroom substrate could substantially enhance its feeding value to be equivalent to PG as an effective fiber source for dairy goat. The fermented rice straw is recommended to account for 15% in diet dry matter.

## INTRODUCTION

Rice comes second to wheat as the main food crop around the globe, and 90% of yields are distributed in East and Southeast Asia [1]. The agricultural statistics yearbook from Agriculture and Food Agency, Council of Agriculture, Taiwan, indicates that the planted areas of rice are about 27.47 million hectares each year, and it is estimated that about five to six tons of rice straw are produced in each hectare [2]. Therefore, the production of rice straw can reach 150 million tons a year. Rice straw is abundant but with poor quality for feed. It contains 25% to 45% cellulose, 18% to 30% hemicellulose (HC), and 10% to 15% lignin [1]. Traditionally, it is a feed ingredient for low production ruminants [1,3]. There are many studies have been conducted on the various physical, chemical, yeast, and white-rot fungi treatments to improve rice straw as ruminant feed [4–7]. Nowadays, most farmers will chop short the straw after harvesting and plow it into the ground. However, the degradability of the fiber in rice straw under natural conditions is slow; moreover, the high production of methane from the degradation process of microbes in the soil under flooding will not only affect the timing of the second crop season but also increase the severity of global warming [2,8–10].

Edible mushrooms can secrete enzymes, including laccases, manganese peroxidase, and lignin peroxidase to decompose lignin [11,12]. From a previous *in vitro* study in our lab, rice straw solid-fermented by *Pleurotus ostreatus* and *Pleurotus sajor-caju* showed the better lignin decreasing efficiency among six selected spent mushroom substrates [13]. Commercial mushrooms are produced on lignocellulosic material. Sawdust is the major substrate used for mushroom cultivation, and over 35 million tons of sawdust are consumed each year in Taiwan. The estimated biological conversion efficiency of substrate for mushroom cultivation is lower than 40%, with 20 million tons of spent mushroom substrate being produced annually [14]. This has caused an urgent problem for farmers in dealing with large amounts of mushroom waste substrate.

The objectives of this study were to determine the effectiveness when combining the two crop byproducts, spent *Pleurotus sajor-caju* mushroom substrate (including mycelia) and rice straw. By using the lignin-digesting enzymes from spent substrate to ferment rice straw and decompose its lignocellulose, it is expected to increase the utility of rice straw as feed for ruminants.

## MATERIALS AND METHODS

Three experiments were conducted in this study, fermented rice straw by spent *Pleurotus sajor-caju* mushroom substrates (FRS) *in situ* degradation, milking performance and rumen degradation of dairy goats fed FRS diet. Fistulated Holstein dry cows, Alpine lactating goats, and Alpine fistulated dry goats involved were raised in the experimental barn at the Livestock Research Institute (LRI) in Taiwan. All experimental procedures were approved by the Institutional Animal Care and Use Committee (IACUC No. 2017-020) of LRI.

### Preparation of fermented rice straw

Dry rice straw (*Oryza sativa* L.) was bought once from a rice straw handling factory in central Taiwan. The variety was unknown, and Tainan 11 rice variety was widely cultivated in Taiwan. It was chopped into 5 to 10 cm in length and put into sacks (2 kg per sack). The sacks were sterilized for one hour in boiling water, spun four minutes in a spin dryer, and put into polyethylene (PE) bags to cool overnight. Next day, 400 g of spent *Pleurotus sajor-caju* mushroom substrate were inoculated with sterilized rice straw (RS) in the PE bags and then sealed and fermented at 25°C to 30°C for eight weeks in a dark room [15].

### *In situ* degradation study

To evaluate the improving effect of fungi fermentation of RS, three rumen fistulated Holstein dry cows were used. They averaged 580 kg body weight and raised with shade and exercise field. Pangolagrass hay and water were provided *ad libitum*. Cows received total 3 kg of grain mixture per day at 8 am and 4 pm. Eight g of tested three forages, pangolagrass hay (PG), RS, and FRS, were weighed into polyester bags, and incubated in the rumen according to Chiou et al [16]. The sample bags (three replicates per forage per hour) were placed into big laundry bags in ventral rumen in reverse order from 96 to 0 hours.

### Milking performance study

To test the benefit of fungi fermentation of RS, diets including individually tested forage were formulated. A total of 21 Alpine goats with milk yield 2.99±0.48 kg and days in milk 137±88 day were randomly assigned into three groups in a 28-day feeding trial. Diet nutrient supply fit the requirements for lactating goats with 3.0 kg of milk yield and 60 kg of body weight according to the NRC [17]. Control diet comprised PG (15% of diet dry matter [DM]), corn silage, alfalfa pellets, byproducts, and grain mixture (Table 1). The FRS and RS were directly substituted for PG in the control diet to form the two treatment diets. There was no nutrition adjustment among diets. Goats were raised in individual high-bed pens (265 cm×110 cm) with their own troughs and water bowls. Diet and water were provided *ad libitum*. Goats were milked twice per day, 7 am and 3 pm, and the total mixture rations were formulated daily and fed twice per day. The first two weeks were an adaptation period for the goats, and trait measurements were executed during the third and fourth weeks. After the first 28-day trial was conducted, the available goats in the barn with similar milk yield and days in milk were selected and assigned to repeat the feeding trial once.

Diet sample from each group was collected four consecutive days during the 3rd and 4th weeks. Eight diet samples each group were pooled, dried, and analyzed for the content of DM, crude protein (CP), crude fat (ether extract, EE), crude ash, calcium (Ca), and phosphorus (P) according to AOAC methods [18] and the neutral detergent fiber (NDF), acid detergent fiber (ADF), and acid detergent lignin (ADL) by using the ANKOM^200^ Fiber Analyzer (Ankom Technology Corp., Fairport, NY, USA). The *in vitro* dry matter digestibility (IVDMD) was analyzed using the Ankom DaisyII system (Ankom Technology Corp., USA) according to Lee and Shiao’s modified method [19]. It consists of the first 48-h rumen microbial fermentation (1 rumen fluid: 4 artificial saliva (v/v)) and the second 24-h pepsin digestion (6% of pepsin). PG, FRS, and RS were sampled eight days and pooled into one each trial. Total two samples each were analyzed as diet samples. Orts of individual goats during the eight-day diet sampling period were also collected to measure the amount and DM percentages to calculate the DM intake of each goat.

Body weights of goats were recorded at the beginning and end of each experiment period. The individual goat milk yield was collected and analyzed two days before the trial started and used as the covariate data for lactating performance statistical analysis. Individual milk yield was recorded four consecutive days during the 4th week. Individual milk samples without preservative were collected three consecutive days and express delivered to analyze the milk composition and somatic cell count (Combi-Foss 5000, Hilleroed, Denmark). At the beginning and end of the replicate trials, 10 mL of blood samples were taken before am-feeding from jugular vein individually. The serum was centrifuged (2,000×g for 20 minutes at 4°C) and stored in a −20°C freezer for analysis. Content of glutamic oxaloacetic transaminase (GOT), glutamic pyruvic transaminase (GPT), total protein (TP), albumin (ALB), globulin, creatinine, total cholesterol (T-Chol), and triglyceride (TG) were analyzed using an automatic analyzer (Hitachi 7176A; Hitachi, Tokyo, Japan) [20].

### Rumen digestion study

Fermented RS, RS, and PG were formulated into three treatment diets and fed to Alpine goats. To monitor the digestion improving potential of FRS diet in rumen, a 3×3 Latin square with three fistulated Alpine dry goats was arranged. The goats were provided three treatment diets sequentially and *ad libitum*. The animal care and management were the same as the dairy goat trial, except they were fed at 8 am once a day. The beginning 12 days let goats adapt to diet, and rumen content was collected at 12 time points: 8:00 (0 hours before feeding), 9:30, 11:00, 13:00, 15:00, 17:00, 19:00, 20:30, 22:00, 00:00 (midnight), 3:00, and 6:00 during the last two days. The filtrated rumen liquid was measured immediately for pH and then acidified and frozen for ammonia nitrogen (NH_3_-N) [21], and volatile fatty acid (VFA) analyses. The VFA was analyzed by GC/FID (CP-3800, Varian) with a 30 m × 0.25 mm × 0.2 μm fused silica capillary column (#24107, Supelco) and a Volatile Fatty Acid Mix analytical standard (Supelco 46975U). The individual VFA were summed up to represent the total volatile fatty acid (TVFA) [22].

### Statistical analysis

The *in situ* degradation study estimates the components of every sample at disappearance timing by the iterative least squares procedure (SAS 9.4; SAS Institute Inc., Cary, NC, USA). According to the model mentioned by Ørskov and McDonald [23], rumen degradation parameters A (readily degraded fraction, %), B (potentially degradable fraction, %), and C (degradation rate of B, %/h, kd) were calculated. The rumen passage rate (kp) was set at 2%/h for NDF and ADF and 5%/h for DM and CP to calculate their effective degradability (ED) in rumen, in which ED = A+B×kd/(kd+kp).

The milking performance and blood biochemistry were analyzed by complete randomized design (CRD) with covariance analysis to eliminate the individual differences of the goats. The data of body weight, diet intake, and rumen digestibility adopted the CRD model. The data from the trial were analyzed by the general linear model. If the analysis of variance showed a significant difference, the least squares mean was used to compare the difference between treatments. The significant level was set at p<0.05, and p<0.10 was also marked and discussed as a trend toward significance.

## RESULTS

### Chemical compositions of the fermented rice straw

The nutrient compositions of the PG, FRS, and RS are shown in Table 2. The ADF, ADL, and crude ash content of the RS were significantly higher than those of the PG, but the HC of the RS was lower (p<0.05). Fungi fermentation seemed to increase CP content of FRS. Compared with RS and PG, FRS significantly alleviated the concentration of NDF and HC, and increased the ADF and Ca concentrations (p<0.05). Lignin content was marginally decreased after fermentation, IVDMD of FRS was elevated to close to PG and tended to be higher than RS (p = 0.11). In addition, the Ca content in the FRS was higher than that in the RS, because CaCO_3_ was added to regulate the pH value of the mushroom substrate.

### *In situ* degradation study

**Figure 1:**
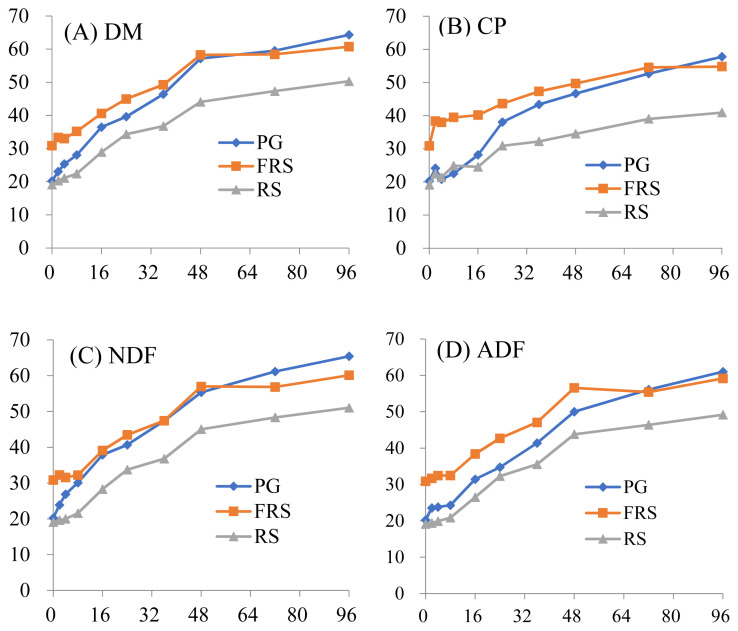
*In situ* disappearances of nutrient components of PG, FRS, and RS tested forages studied with Holstein dry cows. (A) DM, dry matter; (B) CP, crude protein; (C) NDF, neutral detergent fiber; and (D) ADF, acid detergent fiber. (□) PG, pangolagrass hay; (■) FRS, fermented rice straw by spent *Pleurotus sajor-caju* mushroom substrate; and (▲) RS, rice straw. X, hours incubated in the rumen; Y, disappearance of components (%). PG, pangolagrass hay; FRS, fermented rice straw by spent *Pleurotus sajor-caju* mushroom substrate; RS, rice straw.

The *in situ* ruminal degradation kinetics of the components of the three forages, PG, FRS, and RS, are shown in Figure 1A–1D. All three forages were continuously degraded with increasing time. Interestingly, the degradation curve of the FRS in the rumen was more flattened after 48 hours of incubation, while the degradation of the PG and RS was still in progress. The degradation curves of DM, CP, NDF, and ADF of FRS were close to those of the PG and higher than those of the RS. The *in situ* degradability parameters are listed in Table 3. The degradation rates (kd) of four compositions among three forages was similar. The readily degradable parts (A) of DM, CP, NDF, and ADF of FRS were significantly increased compared to RS and PG (p<0.05). The degradable part (A+B) of DM, CP, NDF, and ADF of FRS was higher than RS but lower than PG. The ED of DM, CP, and ADF three compositions of FRS were higher than those in PG and RS (p<0.05). The rumen availability (ED) of NDF in FRS and PG were close and both were higher than RS (p<0.05). The result from *in situ* measurements showed that mycelia of *Pleurotus sajor-caju* could elevate nutrient availability of rice straw in the rumen, which might be due to its enzyme effectively loosening the structure of rice straw.

### Milking performance study

Effects of three forage diets on milking performance in Alpine goats are presented in Table 4. The finding in this study revealed that daily DM intake in FRS group was improved to close to the PG group and higher than the RS group. Fungi fermentation effectively elevated the diet consumption. Compared to RS group, feed intake of FRS group increased more than 20% (2.28 vs 1.81 kg/d, p<0.05). Milk yield of both FRS and PG groups performed better than that of the RS group (3.31 kg vs 3.02 kg/d/goat, p<0.05), with increasing magnitude reached 9%. The 4% FCM (4% fat-corrected milk = [0.4×milk yield] +[15×fat yield]) yield was 3.02 kg in PG group, 2.99 kg in FRS group, and 2.81 kg in RS group. PG group tended to have the higher yield than RS group (p<0.10). Goats fed FRS diet showed higher body weight reservation than goats fed RS diet. Regarding the milk composition, there were no significant differences in protein (average 3.11%), lactose (average 4.16%), and solid-not-fat (average 7.97%) among treatments. However, milk fat and solid-not-fat in the RS group tended to be higher than those in FRS group (p<0.10). Milk urea nitrogen of the PG group tended to be lower than that of the RS group (p<0.10), which showed the carbon-nitrogen balance utility was better for PG diet than that of RS diet. In the daily yield of milk, lactose, solid-not-fat, and total solids, both PG and FRS groups were higher than those in the RS group (p<0.05), which contributed to the higher milk yield.

Effects of forage diets on blood biochemical profile of the lactating goats are listed in Table 5. The GOT of the RS group was significantly higher than that of the PG group (p<0.05), and the value for the FRS group fell between. The GPT, TP, ALB, globulin, creatinine, and T-Chol in each treatment were nearly identical, while TG in PG group tended to be lower than that in RS group (p<0.10).

### Rumen digestion study

**Figure 2:**
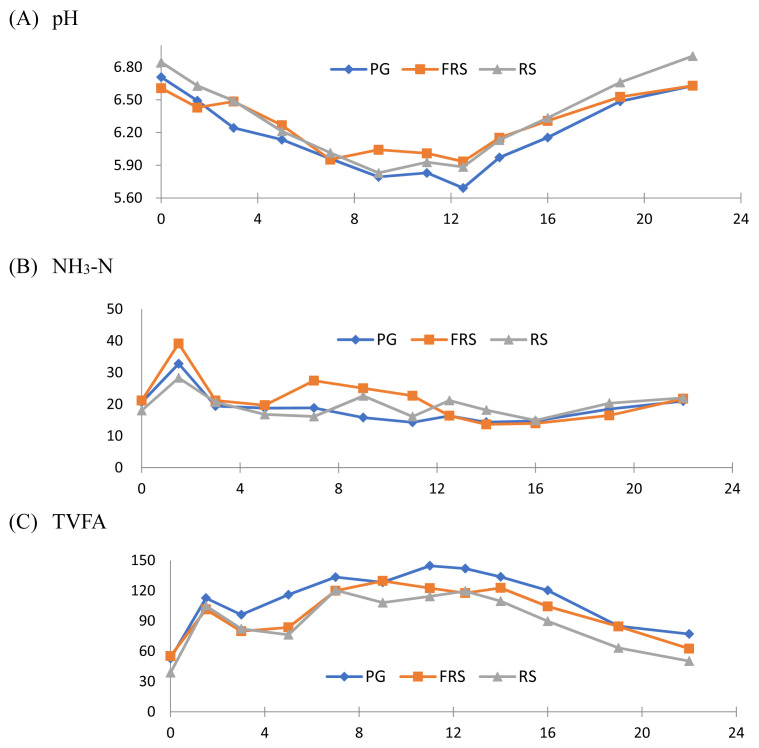
Effect of diets including PG, FRS, or RS on the ruminal pH, NH_3_-N, and TVFA diurnal changes in Alpine dry goats. TVFA: total volatile fatty acid. X: hrs after 8-am feeding, Y: ruminal pH (A), NH_3_-N, mg/dL (B) and TVFA, mM (C). (□) PG, pangolagrass hay; (■) FRS, fermented rice straw by spent *Pleurotus sajor-caju* mushroom substrate; (▲) RS, rice straw. PG, pangolagrass hay; FRS, fermented rice straw by spent *Pleurotus sajor-caju* mushroom substrate; RS, rice straw.

Effects of diets containing PG, FRS, or RS on rumen pH, NH_3_-N, and TVFA diurnal change of Alpine dry goats are shown in Figure 2 and organized in Table 6. The data of the rumen pH, NH_3_-N, individual VFA, and TVFA concentration were shown as weighted average of the 12 samplings. There were no significant differences among the rumen average pH value, the highest, the lowest, and the difference between the highest and the lowest. The pH value dropped to the lowest point after 9 to 12.5 hours of feeding, which was around 8 pm. The average NH_3_-N concentration in the rumen of three groups were similar, between 18.6 and 21.0 mg/dL. The ammonia nitrogen concentration rapidly increased after the morning feeding and then decreased. The daily average butyric acid concentration in the rumen in PG group was higher than the other two groups (Table 6). The propionic acid and TVFA average concentration of goats fed PG diet tended to be higher than RS group (p<0.10). There were no significant differences in the acetic acid, isobutyric acid, isovaleric acid, valeric acid, and caproic acid concentrations among the three groups.

## DISCUSSION

The feeding value of rice straw was improved in this study by the culture of *Pleurotus sajor-caju*. In this study, CP, ADF, and IVDMD of RS were increased while NDF, HC, and ADL decreased after fermentation (Table 2). When the mycelia colonized RS, white-rot fungi secreted enzymes to degrade the lingo-cellulosic complex. The DM digestibility and protein content were determined by the characteristics of the fungi, the composition of the substrate, the temperature, the pH value, and the ventilation during fermentation [24]. Regarding the fiber, the lignin structure changed to an extent that depended on the fermentation process. Most of the white rot fungi would metabolize simple matter, such as hemicellulose (NDF = ADF+HC), before utilizing lignin and cellulose (ADF). Therefore, the residues after fermentation might not be suitable for ruminal microbes to digest [24–26]. Moreover, the metabolites from the lignin fermented by fungi, such as phenolic compounds, inhibited the ruminal microbes and the function of the enzymes in the rumen and caused the decrease of the digestibility rate [25,27].

According to a previous review of white rot fungi, the IVDMD values in the fermented substrate were inconsistent, ranging from 20% enhancement to negative reduction [27]. The main reason was that the fungi decomposed the lignin and cellulose non-specifically. If the fungi belonged to the latter (non-specifically), the decomposing amount of hemicellulose and cellulose would increase, which would then cause the amount of NDF and HC to decrease and the amount of ADF to increase. As a result, the composition of the fermented medium and the characteristics of the white rot fungi would play a key role in the IVDMD results [24,26,28–31]. It was assumed that *Pleurotus sajor-caju* might belong to the latter, not specifically decomposing the lignin and utilize more hemicellulose, which caused the NDF and HC content in the FRS to significantly decrease and the ADF content to relatively increase.

Relative *in situ* studies of the RS DM indicated the readily degraded fraction, potentially degradable fraction, and degradable rate were 11–17.6%, 39–68.8%, and 1.66–5%/h, respectively [8, 32–35], where RS result from this trial were in the range. After fermentation process, the readily degraded fraction of FRS DM increased 66%, from 18.3% to 30.3%, but there were no differences in the potentially degradable fraction or the degradation rate between FRS and RS. The NDF of the FRS content was decreased from 72.48% of RS to 60.29% (Table 2), and the non-fiber carbohydrates (NFC = 100–CP–NDF–EE–crude ash, e.g., starch, glucose, and pectin) of the FRS increased from 5.53% of RS to 14.14%, which supported the dramatic increase in the easily digestible part of the FRS DM. Under a 5% solid passage rate, it was estimated that the ED of the RS DM was 29.48% (Table 3), which was between the result of 27.4% in bulls from Yang et al [35] and 34.6% in Holstein cows from Myung and Kennelly [36]. The FRS after fermentation could significantly elevate its DM degradability in the rumen, and ED5 elevated to 41.67% (about 1.41 folds). Kim et al [32] separated rice straw (fermented) from an *Agaricus bisporus* medium and compared it with the original rice straw (unfermented). The rumen ED increased from 28.7% to 45.1% after fermentation (1.57 times). The solid fermentation of the rice straw with spent *Pleurotus sajor-caju* mushroom substrate in this study showed the similar result as that of Kim et al [32].

The degradation rate of NDF from five strains of RS was between 2.12 and 3.38%/h, and between 2.29 and 3.91%/h for ADF as stated by Ibrahim et al [37], and there was a significant difference between strains of RS. The degradation rate of NDF and ADF of RS were 2.02 and 1.90%/h, respectively, which indicated the RS in this study was degraded slower. In Yang et al [35] bull *in situ* measurement, the rice straw’s ED for NDF and ADF was both 19.1% when kp was set as 5%/h. Myung and Kennelly [36] used non-lactational Holstein cows to perform an *in sacco* measurement, and the ED5 were found to be 20.1% in the NDF and 15.6% in the ADF. In Table 3, the ED for NDF and ADF in the rumen were calculated under 2%/h kp. If the kp changed to 5%/h, ED5 of RS was 16.8% in the NDF and 16.3% in the ADF, which resembled the results with above studies. In this study, the ED of NDF of the RS increased from 27.1% to 37.6% after fermentation, while that for the ADF increased from 26.1% to 37.2%. Following the discussion in previous paragraph regarding composition changes between RS and FRS, it is postulated that the fungi fermentation could not only decompose the lingo-cellulosic linkage but might also partially loosen the cellulose structure of the RS. Results of the *in situ* measurement indicated that rice straw fermented with spent *Pleurotus sajor-caju* mushroom substrate could effectively promote its nutrient availability in the rumen.

That the low feeding value of straw could be enhanced by fungi fermentation was demonstrated in the present study and relative research. Fazaeli et al [38] incubated *Pleurotus cystidiosus* fungi on straw and harvested it twice to partially replace alfalfa in diet for late lactation Holstein cows. The result showed that after supplementing 30% of CP in diet from alfalfa, the intake, fat-corrected-milk yield, or milk compositions were not affected among treatments. After further analyzing the results based on the use of unfermented straw and fermented straw with harvesting mushroom, it was found that the fermented straw could effectively elevate the intake, fat-corrected-milk yield, and milk fat percentage of cows [39]. Meanwhile, three fistulated and castrated bulls were used to compare the unharvested, fungi-harvested, and unfermented wheat straw [40]. The results showed that the total tract digestibility of the straw fermented with unharvested mushrooms was the highest, followed by the straw fermented with harvested mushroom. The lowest digestibility was found with the unfermented straw. The DM intake, organic matter, and intake of digestible organic matter were higher with the fungi-fermented straw. The results for the bulls and dairy cows showed that the fungi-fermented straw could partially replace common forage [38–40]. In our study, feeding diet with FRS by spent *Pleurotus sajor-caju* mushroom substrate could effectively elevate the intake, milk yield, and body weight gain of lactating dairy goats comparing to the goats fed original RS diet, and contribute to the similar performance with goats fed PG diet (15% of diet DM). This result was in accordance with the relative studies.

In this trial, GPT, ALB, TP, globulin, creatinine, T-Chol, and TG concentrations were similar among treatments, and the values all fell in normal range. However, GOT of RS group was significantly higher than those goats fed PG diet (Table 5). Even the GOT value still fell in the normal range (122 to 321 U/L) [20], this phenomenon is worthy to follow-up tracing. Marutsova and Binev [41] study showed that short-time feed deficiency could provoke a reversible microvesicular degeneration of the liver. The increased activity of enzymes GOT and GPT responded the subclinical ketosis and liver parenchyma damage. Oh et al [42] supplemented spent *Pleurotus eryngii* and *Pleurotus ostreatus* mushroom substrates in diet for Hanwoo steers. The blood glucose, ALB, and TP were similar and in the normal range. In a dairy goat trial conducted by Kholif et al [43], mushroom-cultivated straw was used to replace 28% of Egyptian clover for feeding. The ALB, globulin, NH3-N, and creatinine concentrations in the blood all fell in the normal range. The authors concluded that the straw cultivated by mushroom medium would not cause liver damage, abnormal kidney function, or the decomposition of muscle protein.

The easily digested carbohydrates such as sugar and starch are low in the low-quality feedstuff like RS. And these carbohydrate components decrease easily during the fermentation with white-rot fungi, which in turn stabilizes the pH value in the rumen after feeding. The variation of rumen pH of goats fed FRS diet was stable and between the RS group and PG group (Table 6; Figure 2). The NH_3_-N concentration at each sampling time was higher than 5 mg/dL (Figure 2), that microbial synthesis was not limited by the ammonia nitrogen concentration for dairy cows [44]. In this study there was a trend of the propionic acid and TVFA in the goats fed RS diet being lower than the goats fed PG diet (p<0.10), and goats fed FRS diet was between. This higher propionic acid and TVFA indicated the fermentation process could increase the utility of RS in the rumen. Other than the butyric acid concentration of two straw groups being lower than the PG group, the rest metabolites produced in the rumen of PG and FRS groups were numerically higher than those in RS group, implying both the PG and FRS diets could elevate the nutrient supply and reflect on the higher milk yield. Tripathi et al [24] fed mustard straw to sheep and found no significant differences in the pH value, ammonia nitrogen, or TVFA in the rumen. However, concentrations of some enzymes involving digestion decreased sharply, as the fermented mustard straw decreased the number of rumen protozoa. Another study conducted by Karunanandaa and Varga [30] indicated even supplementing adequate protein to the diet, the nitrogen utility of ruminal microbes was weakened if fungi-fermented feed was provided. The adverse effect from fungi-fermented feed in relative study was not seen in this trial as previous study [45,46].

## CONCLUSION

This study found that the spent *Pleurotus sajor-caju* mushroom substrate could effectively improve the nutrient compositions, availability in the rumen, and feeding value of RS. Effective degradability of DM, CP, NDF, and ADF of FRS in the rumen were all significantly increased. In addition, the intake and milk yield of goats are improved by feeding FRS without affecting the health and rumen digestibility of dairy goats. It is suggested that FRS could be an alternative fiber source for dairy goat. The recommended addition could be up to 15% of the dietary DM by replacing pangolagrass hay. The value-added FRS and spent mushroom substrate would not only increase feed supply but also be helpful in resolving the environmental issues for mushroom and rice industry. In the future, further efforts are required to explore the development of a practical fermentation process.

## Figures and Tables

**Table 1:** Diet formula and compositions of FRS offered to Alpine goats (%, DM basis)

Items	Diet treatments		

	PG diet^1)^	FRS diet^1)^	RS diet^1)^
Ingredients			
Pangolagrass hay	15.00	-	-
Fermented rice straw	-	15.00	-
Rice straw	-	-	15.00
Corn silage		10.20	
Dehydrated alfalfa pellet		10.05	
Wet brewers’ grains		12.00	
Soybean hull pellet		12.35	
Wheat bran		5.30	
Grain mixture^2)^,^3)^		35.10	
Diet compositions (analyzed values)			
Dry matter	55.42	46.30	56.22
Crude protein	19.84	20.29	20.01
Neutral detergent fiber	43.26	41.83	43.66
Acid detergent fiber	25.83	27.78	27.43
Hemicellulose^4)^	17.43	14.05	16.22
Acid detergent lignin	2.63	2.66	2.79
Crude fat	3.83	3.71	3.77
Crude ash	7.63	8.92	8.65
Calcium	0.87	0.94	0.87
Phosphorus	0.44	0.45	0.44
*In vitro* dry matter digestibility	68.67	68.59	67.23

DM, dry matter; CP, crude protein.

1) PG, pangolagrass hay; FRS, fermented rice straw by spent *Pleurotus sajor-caju* mushroom substrates; RS, rice straw.

2) Grain mixture was same for three groups, including ground corn 57.50%, soybean meal (CP 43%) 29.20%, fish meal (CP 60%) 3.10%, molasses 2.50%, salt 1%, limestone 2%, dicalcium phosphate 0.4%, potassium carbonate 1%, sodium bicarbonate 1.5%, urea 1%, vitamin premix 0.60%, and mineral premix 0.20%. (as fed basis).

3) Each g of vitamin premix provided 10,000 IU of vitamin A, 2,000 IU of vitamin D_3_, and 55 IU of vitamin E. Each kg of mineral premix provided 16 g of Cu, 6 g of Mn, 0.2 g of Co, 30 g of Zn, 1.5 g of I, and 0.3 g of Se.

4) Hemicellulose = neutral detergent fiber – acid detergent lignin.

**Table 2:** Nutrient compositions and in vitro DM digestibility of PG, FRS, and RS (%, DM basis)

Items	Forages^1)^			SEM	p-value

	PG	FRS	RS		
CP	4.98^b^	9.28^a^	7.12^ab^	0.61	0.04
NDF	69.83^a^	60.29^b^	72.48^a^	1.51	0.02
ADF	39.26^c^	52.30^a^	50.00^b^	0.10	0.01
HC^2)^	30.57^a^	7.99^c^	22.48^b^	1.59	0.02
ADL	3.81^b^	4.07^ab^	4.88^a^	0.29	0.05
EE	1.56^a^	0.80^b^	1.15^ab^	0.12	0.05
Crude ash	6.85^b^	15.49^a^	13.72^a^	0.61	0.01
Ca	0.33^b^	0.81^a^	0.37^b^	0.06	0.02
P	0.10^b^	0.20^a^	0.14^ab^	0.01	0.05
IVDMD^#^	51.80^A^	51.28	42.13^B^	2.82	0.15

SEM, standard error of mean; DM, dry mater; CP, crude protein; NDF, neutral detergent fiber; ADF, acid detergent fiber; HC, hemicellulose; ADL, acid detergent lignin; EE, ether extract; Ca, calcium; P, phosphorus; IVDMD, in vitro dry matter digestibility.

1) PG, pangolagrass hay; FRS, fermented rice straw by spent Pleurotus sajor-caju mushroom substrates; RS, rice straw.

2) HC = NDF–ADF.

a–c Means in the same row with different superscripts differ significantly (p<0.05).

A,B Indicates p<0.10.

# PG>RS (p = 0.09); FRS>RS (p = 0.11).

**Table 3:** *In situ* degradation parameters of nutrient components of PG, FRS, and RS in Holstein dry cows

Items	Parameters	Forages^1)^			SEM	p-value

		PG	FRS	RS		
DM	A	21.07^b^	30.32^a^	18.30^c^	0.39	0.01
	B	51.54^a^	34.82^b^	39.23^b^	3.02	0.02
	kd	1.97	2.42	2.18	0.37	0.70
	ED5	35.34^b^	41.67^a^	29.48^c^	0.25	0.01
CP	A	32.28^b^	52.59^a^	26.80^c^	0.44	0.01
	B^#^	43.93^A^	17.03^B^	36.01	8.41	0.15
	kd	1.77	2.23	1.84	0.68	0.88
	ED5	42.70^b^	57.79^a^	32.97^c^	0.32	0.01
NDF	A	7.18^b^	15.30^a^	3.25^c^	0.54	0.01
	B	60.96^a^	42.89^b^	49.97^ab^	3.70	0.04
	kd	2.17	2.17	2.02	0.36	0.95
	ED2	38.11^a^	37.57^a^	27.10^b^	0.37	0.01
ADF	A	4.77^b^	15.90^a^	3.74^b^	0.34	0.01
	B	61.73^a^	40.89^b^	49.14^ab^	4.58	0.05
	kd	1.72	2.18	1.90	0.37	0.70
	ED2	33.02^b^	37.18^a^	26.11^c^	0.41	0.01

SEM, standard error of mean; DM, dry matter; A, readily degraded fraction (%); B, potentially degradable fraction (%); kd, degradation rate of B (%/hr); ED5, effective degradability of ruminal solid passage rate (kp) set as 5%/h; CP, crude protein; ED2, effective degradability of ruminal solid passage rate (kp) set as 2%/h; NDF, neutral detergent fiber; ADF, acid detergent fiber.

1) PG, pangolagrass hay; FRS, fermented rice straw by spent *Pleurotus sajor-caju* mushroom substrates; RS, rice straw.

a–c Means in the same row with different superscripts differ significantly (p<0.05).

A,B Indicates p<0.10.

# PG>FRS (p = 0.07).

**Table 4:** Effect of dietary inclusion of PG, FRS, or RS on milking performance of Alpine dairy goats

Items	Diet treatments^1)^			SEM	p-value

	PG diet	FRS diet	RS diet		
Dry matter intake (kg/d)	2.21^a^	2.28^a^	1.81^b^	0.09	0.01
Milk production (kg/d)	3.28^a^	3.33^a^	3.02^b^	0.07	0.01
4% FCM (kg/d) ^&^	3.02^A^	2.99	2.81^B^	0.08	0.14
BW change (g/d)	45^ab^	84^a^	9^b^	18	0.02
Milk fat (%)^*^	3.49	3.34^B^	3.54^A^	0.08	0.19
Milk protein (%)	3.11	3.09	3.12	0.02	0.63
Milk lactose (%)	4.21	4.13	4.15	0.03	0.25
Solid not fat (%)	8.01	7.92	7.98	0.05	0.43
Milk total solid (%)^*^	11.49	11.26^B^	11.55^A^	0.11	0.14
MUN (mg/dL)^#^	28.92^B^	31.05	31.17^A^	0.84	0.12
Calculated yields of milk compositions (g/d)					
Milk fat	114	111	107	4	0.35
Milk protein	102	102	95	4	0.46
Milk lactose	138^a^	137^a^	126^b^	3	0.02
Solid not fat	262^a^	263^a^	241^b^	6	0.03
Milk total solid	376^a^	374^a^	348^b^	9	0.04

SEM, standard error of mean; 4% FCM, 4% fat-corrected milk; BW, body weight; MUN, milk urea nitrogen.

1) PG, pangolagrass hay; FRS, fermented rice straw by spent *Pleurotus sajor-caju* mushroom substrates; RS, rice straw.

a,b Means in the same row with different superscripts differ significantly (p<0.05).

A,B Indicates p<0.10.

& PG diet>RS diet, p<0.10.

* RS diet>FRS diet, p<0.10.

# RS diet>PG diet, p<0.10.

**Table 5:** Effect of dietary inclusion of PG, FRS, or RS on blood biochemical profile in Alpine lactating goats

Items	Diet treatments^1)^			SEM	p-value

	PG diet	FRS diet	RS diet		
GOT (U/L)	122^b^	141^ab^	150^a^	9	0.04
GPT (U/L)	15.68	16.16	16.24	0.53	0.74
TP (g/dL)	7.75	7.69	7.76	0.09	0.82
ALB (g/dL)	4.14	4.11	4.13	0.04	0.83
Globulin (g/dL)	3.59	3.61	3.62	0.07	0.93
Creatinine (mg/dL)	0.81	0.83	0.80	0.01	0.92
T-Chol (mg/dL)	123	120	120	3	0.78
TG (mg/dL)^#^	8.57^B^	9.83	10.96^A^	0.85	0.06

SEM, standard error of mean; GOT, glutamic oxaloacetic transaminase; GPT, glutamic pyruvic transaminase; TP, total protein; ALB, albumin; T-Chol, total cholesterol; TG, triglyceride.

1) PG, pangolagrass hay; FRS, fermented rice straw by spent *Pleurotus sajor-caju* mushroom substrate; RS, rice straw.

a,b Means in the same row with different superscripts differ significantly (p<0.05).

A,B indicates p<0.10.

# PG diet<RS diet, p<0.10.

**Table 6:** Effect of dietary inclusion of PG, FRS, or RS on rumen degradation in Alpine dry goats

Items	Diet treatments^1)^			SEM	p-value

	PG	FRS	RS		
Diurnal wei. pH	6.20	6.30	6.35	0.08	0.38
Highest pH (H)	6.71	6.63	6.90	0.15	0.65
Lowest pH (L)	5.69	5.94	5.83	0.10	0.75
H–L	1.02	0.69	1.07	0.14	0.84
Diurnal wei. NH_3_-N (mg/dL)	18.57	21.01	19.44	1.32	0.33
Diurnal wei. VFA (mM)					
C_2_	72.07	66.64	58.78	5.21	0.31
C_3_^*^	22.23^A^	18.27	16.60^B^	1.67	0.19
Iso-C_4_	0.91	0.94	0.93	0.06	0.98
C_4_	12.17^a^	8.98^b^	8.57^b^	0.75	0.05
Iso-C_5_	1.01	1.12	1.09	0.12	0.78
C_5_	1.14	1.04	0.94	0.09	0.50
C_6_	0.24	0.20	0.20	0.04	0.76
Total VFA ^*^	109.76^A^	97.18	87.12^B^	7.08	0.19

Values were expressed as 24-hr diurnally weighed average.

SEM, standard error of mean; VFA, volatile fatty acid; C_2_, acetic acid; C_3_, propionic acid; Iso-C_4_, isobutyric acid; C_4_, butyric acid; Iso-C_5_, isovaleric acid; C_5_, valeric acid; C_6_, caproic acid; Total VFA, summed of individual VFAs.

1) PG, pangolagrass hay; FRS, fermented rice straw by spent Pleurotus sajor-caju mushroom substrate; RS, rice straw.

a,b Means in the same row with different superscripts differ significantly (p<0.05).

A,B indicates p<0.10.

* PG diet>RS diet, p<0.10.
